# Air Pollution and ASDs: Homing In on an Environmental Risk Factor

**DOI:** 10.1289/ehp.123-A68

**Published:** 2015-03-01

**Authors:** Carrie Arnold

**Affiliations:** Carrie Arnold is a freelance science writer living in Virginia. Her work has appeared in *Scientific American*, *Discover*, *New Scientist*, *Smithsonian*, and more.

Although researchers have begun making significant inroads into understanding the genetic and biological basis for autism spectrum disorders (ASDs) and other neurodevelopmental disorders, it’s been estimated that environmental factors could account for just over half the risk of developing ASDs.^1^ In this issue of *EHP*, researchers have used data from 1,767 women in the Nurses’ Health Study (NHS) II to study exposure to particulate matter (PM) as one potential environmental risk factor for ASDs.^2^

In one of the first reports of a relationship between air pollution and ASDs, investigators in California found associations between estimated exposures to airborne heavy metals and other pollutants and risk of ASDs.^3^ Later studies using the same exposure models as the California study reported links between multiple hazardous air pollutants and ASDs in North Carolina and West Virginia,^4^ and a U.S.-wide study had similar findings.^5^ Other work considered how close pregnant women lived to freeways,^6^ or used monitor-based estimates of air pollutants^7^^,^^8^^,^^9^ or models of traffic-related exposures to study links with ASD risk.

**Figure:**
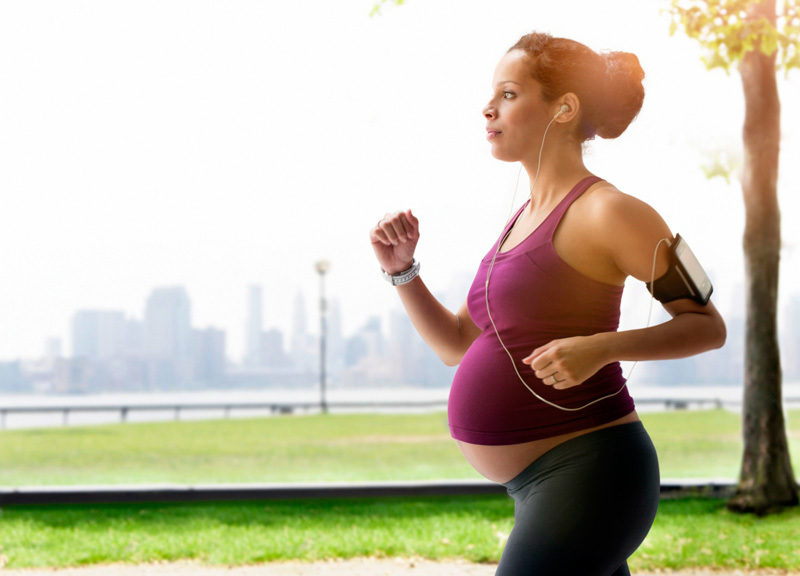
In this study, higher exposures to PM_2.5_ air pollution during the third trimester of pregnancy were associated with increased risk of having a child with an autism spectrum disorder. © KidStock/Blend Images/Corbis

“All these studies seemed to be circling around different aspects of air pollution but showing similar signals,” says Marc Weisskopf, an epidemiologist at Harvard and senior author of the current study. “We wanted to look at this in a U.S.-wide population with better models and with more precision.”

The current study included 245 mothers of children with ASDs and 1,522 controls. The researchers used each mother’s mailing address from before, during, and after pregnancy to estimate her exposure to airborne coarse and fine particulate matter (PM_10_ and PM_2.5_, respectively). Spatiotemporal models took into account data from the U.S. Environmental Protection Agency’s Air Quality System and other monitors along with geographic predictors such as proximity to major roads to estimate exposures on a monthly basis at the nurses’ residences.^10^^,^^11^^,^^12^

The coarser fraction of PM showed no clear relationship with ASD risk. However, the researchers estimated 50% higher odds of having a child with an ASD in women in the highest quartile of estimated PM_2.5_ exposure throughout pregnancy, compared with women in the lowest quartile, after controlling for child sex, month and year of birth, maternal and paternal age at birth, and median census tract income. In particular, the researchers found that the association between ASDs and exposure to PM_2.5_ was strongest during the third trimester of pregnancy.^2^

“This study provides some really nice continuing evidence of the potential relationship between air pollution and autism across the entire United States,” says Heather Volk, an epidemiologist at the University of Southern California, who was not involved in the study.

Previous work in animal models has suggested a possible mechanism for how air pollution exposure may increase ASD risk. Mice exposed to concentrated ambient ultrafine PM showed ventriculomegaly, altered neurochemistry, and activation of glial cells in the brain.^13^ These responses, which occurred preferentially in male mice, signal an inflammatory response. Weisskopf says that an inflammatory response in the mother or developing fetus is currently a leading hypothesis for how PM may increase ASD risk.

But any environmental risk, researchers say, almost certainly interacts with genetic factors that either increase or decrease ASD risk. “In a complex disease like autism, it’s not going to be just genes or just environment,” Volk says.
